# Four new species of *Closterocerus* Westwood (Hymenoptera, Eulophidae) from China, with a key to Chinese species

**DOI:** 10.3897/zookeys.1017.62256

**Published:** 2021-02-12

**Authors:** Ming-Rui Li, Cheng-De Li

**Affiliations:** 1 School of Forestry, Northeast Forestry University, Harbin, 150040, China Northeast Forestry University Harbin China

**Keywords:** Chalcidoidea, Entedoninae, natural enemy, parasitoid wasp, taxonomy

## Abstract

Four new species of *Closterocerus* Westwood, *C. rectisulcus***sp. nov.**, *C. shaanxiensis***sp. nov.**, *C. separatus***sp. nov.** and *C. unifasciatus***sp. nov.** are described from China, each with a distinct pattern on the fore wings, and belonging to subgenus Closterocerus. A key to all species of the genus *Closterocerus* in China is provided.

## Introduction

*Closterocerus* Westwood (Hymenoptera, Eulophidae, Entedoninae), contains 74 valid species worldwide, but only four species have been recorded from China (Noyes 2020). Hansson (1994) divided the Nearctic species into two subgenera, *Achrysocharis* Girault and *Closterocerus* Westwood according to whether the pedicel was compressed or not. Species of subgenus Closterocerus, with a more or less compressed pedicel provided with dorsal and ventral edges, include one Holarctic species (*C. trifasciatus* Westwood), seven Nearctic species (*C. brachyphagus* Hansson, *C. cincinnatus* Girault, *C. cinctipennis* Ashmead, *C. nitidus* Hansson, *C. ruforum* (Krausse), *C. tau* Girault, *C. utahensis* Crawford) and one Palearctic species (*C. lyonetiae* (Ferrière)). Additional species from other parts of the world possibly belong to the subgenus Closterocerus, but the descriptions lack the character Hansson (1994) used to characterize the subgenus.

In the present paper, we describe four new species of the subgenus Closterocerus s. str. from China, each with a distinct pattern on the fore wing, and a key to all species of the genus *Closterocerus* distributed in China is provided.

## Material and methods

Specimens were collected by sweeping, and were dissected and mounted in Canada Balsam on slides following the method of Noyes (1982), or mounted on a card. Photos were taken with a digital CCD camera attached to an Olympus BX51 compound microscope or Aosvi AO-HK830-5870T digital microscope. Measurements were made using an eye-piece reticle, or using the ruler tool in Adobe Photoshop 2020.

Terminology follows the Hymenoptera Anatomy Consortium (2020), and the following abbreviations are used:

**F1–5** flagellomeres 1–5;

**HE** height of eye;

**MS** malar space;

**MV** marginal vein;

**OOL** minimum distance between a posterior ocellus and corresponding eye margin;

**PMV** postmarginal vein;

**POL** minimum distance between posterior ocelli;

**SMV** submarginal vein;

**STV** stigmal vein;

**WM** width of mouth opening.

All type material is deposited in the insect collections at Northeast Forestry University (**NEFU**), Harbin, China.

## Taxonomy

### Key to Chinese species of genus *Closterocerus* (females)

**Table:** 

1	Pedicel and flagellum not compressed, and all flagellomeres longer than wide (fig. 1A in Yang and Luo 1994); fore wing without distinct infuscate transverse band, only with a brown spot around STV (fig. 1C in Yang and Luo 1994)	***C. litchii* (Yang & Luo, 1994)**
–	Pedicel and flagellum compressed, and at least 1–2 flagellomeres wider than long or quadrate (e.g. Figs 5, 31); fore wing with 1–3 distinct infuscate bands (Figs 7, 25, 33) (*Closterocerus* s. str.)	**2**
2	Scape slightly compressed, widest in middle part; F3 wider than F2 (Fig. 31)	***C. unifasciatus* Li & Li, sp. nov.**
–	Scape strongly compressed, widest at apex; F3 at most as wide as F2, usually narrower (Fig. 5)	**3**
3	Frontal sulcus V-shaped (Figs 20, 22); infuscate transverse band at apex of fore wing not V-shaped	**4**
–	Frontal sulcus straight (Figs 2, 4); infuscate transverse band at apex of fore wing V-shaped (Fig. 7)	**7**
4	Mesoscutellum distinctly convex, metascutellum small, predominantly hidden under mesoscutellum	***C. cincinnatus* Girault, 1916**
–	Mesoscutellum distinctly flat, metascutellum larger, not hidden under mesoscutellum (Figs 21, 32)	**5**
5	Fore wing, between cubital setal line and hind margin of wing with a longitudinal infuscation (Fig. 25); head in frontal view about 1.4 times as wide as high, nearly oval (Fig. 20)	***C. separatus* Li & Li, sp. nov.**
–	Fore wing, between cubital setal line and hind margin of wing hyaline, without the longitudinal infuscation; head in frontal view about 1.65 times as wide as high, nearly triangular (Fig. 2)	**6**
6	F2 distinctly wider and longer than F1; median part of midlobe of mesoscutum usually differently colored from lateral parts of midlobe; median part of mesoscutellum usually with a differently colored longitudinal band	***C. trifasciatus* Westwood, 1833**
–	F2 similar to F1; mesoscutum and mesoscutellum always unicolorous	***C. eutrifasciatus* Liao, 1987**
7	Propodeal plica absent, spiracular sulcus present (Fig. 6); transverse V-shaped band at apex of fore wing dark and distinct (Fig. 7)	***C. rectisulcus* Li & Li, sp. nov.**
–	Propodeal plica present, spiracular sulcus absent; transverse V-shaped band at apex of fore wing much paler and obscure (Fig. 14)	***C. shaanxiensis* Li & Li, sp. nov.**

#### Closterocerus rectisulcus

Taxon classificationAnimaliaHymenopteraEulophidae

Li & Li sp. nov.

EAA1CA6B-FEEF-57C4-AA9D-1D4761299A99

http://zoobank.org/9DD5A3E2-25AF-4EBD-BB29-56AFDC94F088

Figs 1–3 , 4–9

##### Type material.

***Holotype***: ♀ [NEFU; on slide], China, Heilongjiang Province, Shangzhi City, Maoershan, 04.VIII. 2016, Si-Zhu Liu, Ye Chen and Hai-Yan Wang, sweeping. ***Paratypes***: 4♀ [1 on slide, 3 in alcohol], same data as holotype; 2♀ [in alcohol], China, Liaoning Province, Anshan City, Mountain Qian Shan, 23.VI.2015, Hui Geng, Si-Zhu Liu, Zhi-Guang Wu and Yan Gao, sweeping; 1♀ [in alcohol], China, Shaanxi Province, Ankang City, Ningshan County, Guanghuojie Town, 03.VIII.2015, Ye Chen and Chao Zhang, sweeping; 2♀ [1 on card, 1 in alcohol], China, Shaanxi Province, Ankang City, Ningshan County, Chengguan Town, Huoditang Forestry Station, 11.VIII.2015, Ye Chen and Chao Zhang, sweeping.

**Figures 1–3.**
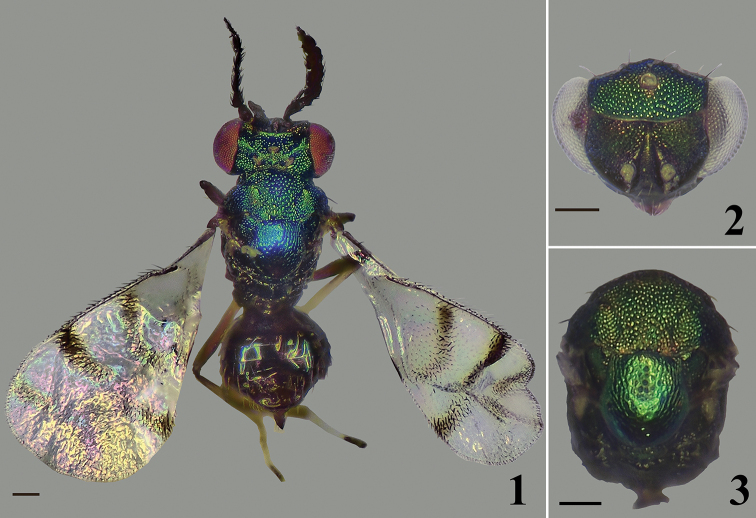
*Closterocerus rectisulcus* Li & Li, sp. nov., paratypes, females **1** habitus in dorsal view, on card **2** head, frontal view, before slide-mounting **3** mesosoma and petiole, before slide-mounting. Scale bars: 100 μm.

##### Diagnosis.

Frontal sulcus straight, reaching eye margins; fore wing with three infuscate transverse bands (Fig. 7): band I V-shaped, with upper arm longer and more distinct than lower arm; band II obscure medially or nearly interrupted; band III V-shaped, with upper arm slightly longer than lower arm; the three bands are nearly connected in the middle; stigmal hairline absent, radial cell setose; below base of cubital setal line with five setae in a row; propodeum with a short median carina delimited by a transverse carina posteriorly; propodeal plica absent; spiracular sulcus present.

**Figures 4–9.**
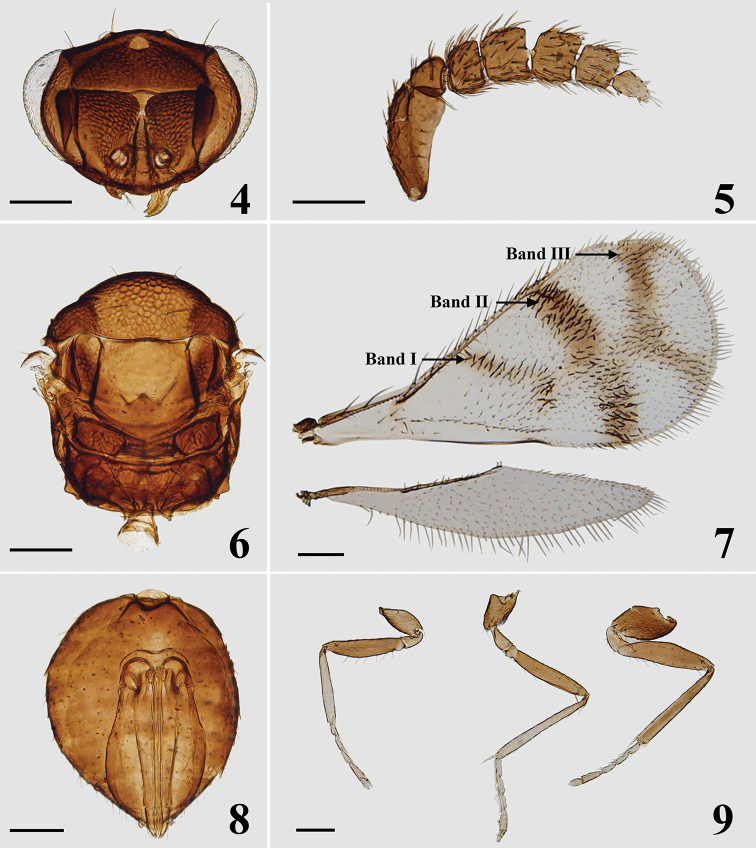
*Closterocerus rectisulcus* Li & Li, sp. nov., holotype, female, on slide: **4** head, frontal view **5** antenna **6** mesosoma and petiole **7** fore and hind wings **8** metasoma without petiole **9** legs, from left to right: fore, mid and hind leg. Scale bars: 100 μm.

##### Description.

**Female.** Length 0.9–1.3 mm. Scape and pedicel dark brown to black. Flagellum dark brown to pale brown, becoming gradually paler distad. Eyes red, ocelli pale brown. Vertex and frons above frontal sulcus golden-green to golden-blue. Frons below frontal sulcus golden-yellow to golden-green. Mandibles pale brown. Pronotal collar, mesoscutum, mesoscutellum and axillae golden-green in dorsal view, golden-blue in lateral view. Metascutellum and propodeum dark bronze. Fore wing (Fig. 7) with three infuscate transverse bands: band I (below the middle of MV) V-shaped, with upper arm longer and more distinct than lower arm; band II (below STV), obscure medially or nearly interrupted; band III (at apical margin of fore wing) V-shaped, with upper arm slightly longer than lower arm; the three bands are nearly connected in middle. All coxae, femora and tarsal claws dark brown (metacoxae darker, nearly black); trochanters pale brown; protibiae and mesotibiae mainly pale yellow, slightly pale brown basally; metatibiae dark brown; pro- and mesotarsi pale yellow with last tarsomere pale brown; metatarsi pale yellow with last tarsomere dark brown. Metasoma dark brown with weak metallic bluish-green to bronze reflections.

***Head*** (Figs 2, 4), in frontal view 1.4 times as wide as high. Sculpture on vertex and frons above frontal sulcus nearly with the same sized meshes. POL : OOL = 7 : 5. Frontal sulcus straight, reaching eye margins; inner eye margins slightly concave in lower part. Antennal scrobes join on frontal sulcus. Malar sulcus absent, but with a curved transverse carina near clypeus, extending to lower eye margins. Clypeus not delimited. HE : MS : WM about 4.8 : 1.0 : 3.3. Antenna (Fig. 5) inserted slightly above level of lower margin of eyes. Scape reticulate, extremely compressed, and expanded from base to apex, about 2.1 times as long as wide. Pedicel moderately compressed compared to the extremely compressed scape, slightly shorter than wide (ca 4 : 5). Flagellum extremely compressed; F1–3 wider than long, F2 longer and wider than F1 and F3; F4 quadrate; F5 small, tapering distad, with terminal spine shorter than the segment.

***Mesosoma*** (Figs 3, 6). Pronotum, mesoscutum, mesoscutellum, axillae and posterior part of propodeum with reticulate sculpture, meshes nearly of same size (but wider on mesoscutellum). Metascutellum with irregular rugae. Pronotum transverse, invisible in dorsal view. Median area of mesoscutum with two pairs of setae. Notauli curved in anterior part, and indicated posteriorly by depression. Mesoscutellum 0.97 times as long as wide. Axillae slightly advanced forwards in front of level of anterior margin of mesoscutellum. Mesoscutum and mesoscutellum slightly convex. Metascutellum about 1/3 as long as median length of propodeum. Propodeum with a short median carina delimited by a transverse carina posteriorly. Propodeal plica absent, spiracular sulcus present. Fore wing (Fig. 7) about twice as long as wide, without stigmal hairline, radial cell setose. Speculum closed below. Ratio length of: SMV : MV : PMV : STV about 3 : 6 : 1 : 1. Cubital setal line straight and completely extending to base of MV. Below base of cubital setal line with five setae in a row. Hind wing (Fig. 7) about 4.5 times as long as wide. Legs (Fig. 9) with all coxae reticulate on outer surface; ventral margin of pro- and metafemur with six and ten long setae respectively; mesotibial spur about 0.9 times as long as corresponding basitarsus.

***Metasoma*** (Fig. 8) ovate; petiole short, pyriform; ovipositor exserted beyond apex of metasoma.

**Male.** Unknown.

##### Host.

Unknown.

##### Etymology.

The name refers to the straight frontal sulcus in this species (*rectus* is Latin for straight).

##### Distribution.

China (Heilongjiang, Liaoning, Shaanxi provinces).

##### Remarks.

*Closterocerus rectisulcus* sp. nov. is similar to *C. orientalis* Yefremova & Kriskovich, 1996 because they share a similar pattern of the fore wing according to the description. The new species differs as follows: pedicel slightly shorter than, or at most as long as wide (longer than wide in *C. orientalis*); mesoscutellum approx. as long as wide (three times as long as wide in *C. orientalis*); mesotibial spur 0.9 times as long as corresponding basitarsus (1.7 times as long as corresponding basitarsus in *C. orientalis*).

#### Closterocerus shaanxiensis

Taxon classificationAnimaliaHymenopteraEulophidae

Li & Li sp. nov.

B255B7A5-2AE0-5E0F-937D-C5D311DC190F

http://zoobank.org/4FBDD51E-135A-4D06-837F-27019927CF25

Figs 10 , 11–18

##### Type material.

***Holotype***: ♀ [NEFU; on slide], China, Shaanxi Province, Ankang City, Ningshan County, Chengguan Town, Huoditang Forestry Station, 11.VIII.2015, Ye Chen and Chao Zhang, sweeping. ***Paratype***: 1♀ [on card], China, Shaanxi Province, Ankang City, Ningshan County, Chengguan Town, Huoditang Forestry Station, 09.VIII.2015, Ye Chen and Chao Zhang, sweeping.

##### Diagnosis.

Frontal sulcus straight, reaching eye margins; fore wing with band I V-shaped, with upper arm much longer and darker than lower arm, the lower arm obscure; band II distinct and interrupted medially; band III V-shaped, obscure, much paler than band I and II, with upper arm slightly longer than lower arm; the three bands are separated from each other; fore wing without stigmal hairline, radial cell setose; below base of cubital setal line with nine setae in a row; propodeal plica present, spiracular sulcus absent.

**Figure 10.**
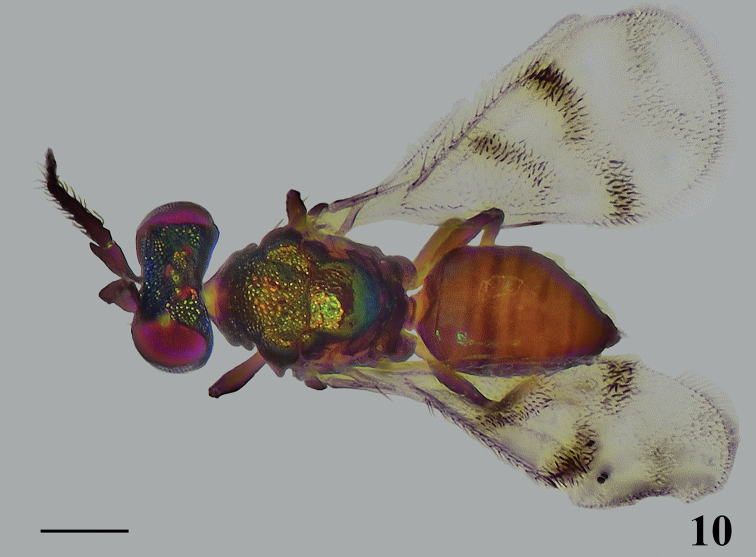
*Closterocerus shaanxiensis* Li & Li, sp. nov., holotype, female, habitus in dorsal view, before dissection. Scale bar: 200 μm.

##### Description.

**Female.** Length 1.1–1.3 mm. Scape and pedicel dark brown to black. Flagellum dark brown to pale brown, becoming gradually paler distad. Eyes red, ocelli pale brown to red. Vertex and frons above frontal sulcus golden-green to golden-blue. Frons below frontal sulcus mainly golden-yellow with golden-green tinge. Pronotal collar, mesoscutum, mesoscutellum and axillae golden-green to golden-yellow in dorsal view, golden-blue in lateral view. Metanotum and propodeum brown, without metallic reflections, except the median part of metascutellum concolorous with mesoscutellum. Fore wing (Fig. 16) with band I V-shaped, with upper arm much longer and darker than lower arm, the lower arm much obscure; band II distinct and interrupted medially; band III V-shaped, obscure, much paler than the band I and II, with upper arm slightly longer than lower arm; the three are separated from each other. Legs with all coxae, femora and tarsal claws dark brown; trochanters pale brown; protibiae mainly pale yellow, pale brown basally; mesotibiae with basal half pale brown and apical half pale yellow; metatibiae dark brown; tarsi pale yellow with last tarsomere pale brown to brown. Metasoma brown with rather weak reflections.

**Figures 11–18.**
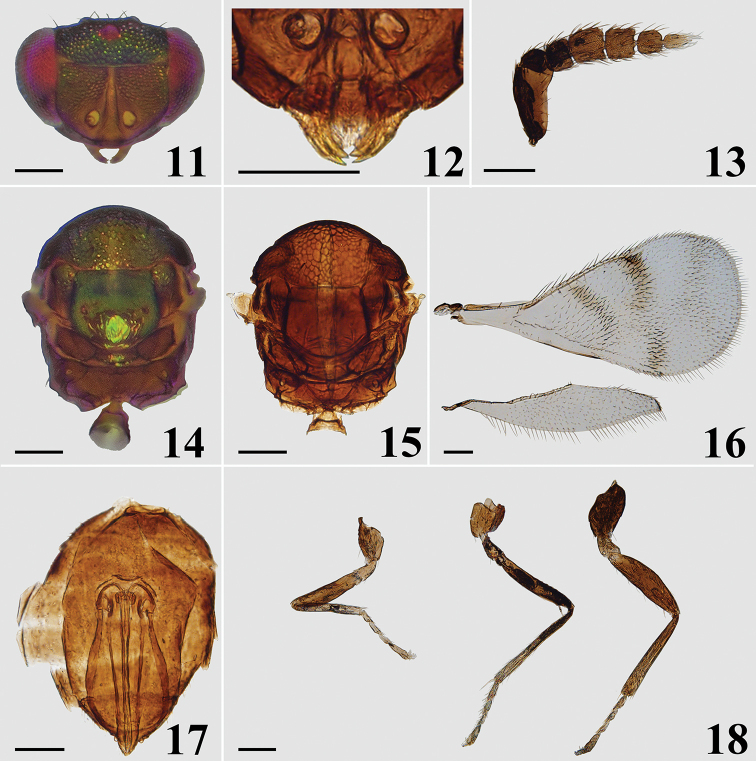
*Closterocerus shaanxiensis* Li & Li, sp. nov., holotype, female, on slide (except **11** and **14**, which are photographed before slide-mounting): **11** head, frontal view **12** mandibles **13** antenna **14, 15** mesosoma and petiole **16** fore and hind wings **17** metasoma without petiole **18** legs, from left to right: fore, mid and hind leg. Scale bars: 100 μm.

***Head*** (Figs 11, 12), in frontal view 1.45 times as wide as high. Sculpture on vertex and frons above frontal sulcus nearly with the same sized meshes. POL : OOL = 6 : 5. Frontal sulcus straight, reaching eye margins; inner eye margins slightly concave in lower part. Antennal scrobes join on frontal sulcus. Malar sulcus absent, but with a curved transverse carina near clypeus, extending to lower margin of eyes. Clypeus not delimited. HE : MS : WM about 3.9 : 1.0 : 2.2. Antenna (Fig. 13) inserted slightly above level of lower margin of eyes. Scape reticulate, extremely compressed, and expanded from base to apex, about 2.2 times as long as wide. Pedicel moderately compressed compared to the extremely compressed scape, slightly longer than wide (about 9 : 8). Flagellum extremely compressed; F1 wider than long; F2 and F3 quadrate; F4 slightly longer than wide; F2 widest and longest and gradually tapering from F2 to F5; F5 with terminal spine shorter than the segment.

***Mesosoma*** (Figs 14, 15). Pronotum, mesoscutum, mesoscutellum and axillae with reticulate sculpture, meshes nearly of same size. Metascutellum with irregular rugae. Pronotum transverse, invisible in dorsal view. Median area of mesoscutal midlobe with two pairs of setae. Notauli curved in anterior part, and indicated posteriorly by depression. Mesoscutellum approx. as long as wide. Axillae slightly advanced forwards in front of level of anterior margin of mesoscutellum. Mesoscutum and mesoscutellum slightly convex. Metascutellum about 3/5 as long as median length of propodeum. Propodeum without reticulate sculpture, with a short median carina delimited by a transverse carina posteriorly. Propodeal plica present, spiracular sulcus absent. Fore wing (Fig. 16) about twice as long as wide, without stigmal hairline, radial cell setose. Speculum closed below. Ratio length of: SMV : MV : PMV : STV about 4 : 8 : 1 : 1. Cubital setal line straight and completely extending to base of MV. Below base of cubital setal line with nine setae in a row. Hind wing (Fig. 16) about 4.5 times as long as wide. Legs (Fig. 18) normal.

***Metasoma*** (Fig. 17) ovate; petiole short, pyriform; ovipositor exserted beyond apex of metasoma.

**Male.** Unknown.

##### Host.

Unknown.

##### Etymology.

The specific name is derived from the name of the collection locality, Shaanxi Province.

##### Distribution.

China (Shaanxi Province).

##### Remarks.

*Closterocerus shaanxiensis* sp. nov. is similar to *C. rectisulcus* sp. nov., but differs as follows: fore wing below base of cubital setal line with nine setae in a row (five in *C. rectisulcus*); propodeum smooth, without reticulate sculpture (with reticulate sculpture posteriorly in *C. rectisulcus*); propodeal plica present (absent in *C. rectisulcus*); spiracular sulcus absent (present in *C. rectisulcus*). The pattern on the fore wing and color of the metasoma are also different from that in *C. rectisulcus*: band III obscure, much paler than band I and II; the three bands are separated from each other (in *C. rectisulcus*, band III distinct, only slightly paler than band I and II; the three bands are nearly connected in the middle); metasoma brown (dark brown in *C. rectisulcus*).

#### Closterocerus separatus

Taxon classificationAnimaliaHymenopteraEulophidae

Li & Li sp. nov.

D77C6AA5-4118-5461-9E8B-E21771712480

http://zoobank.org/6C889A04-1340-47E0-9513-5672DE233418

Figs 19–21 , 22–27

##### Type material.

***Holotype***: ♀ [NEFU; on slide], China, Heilongjiang Province, Hegang City, Wuzhishan Park, 22.VII.2020, Ming-Rui Li, sweeping. ***Paratypes***: 2♀ [on slides], same data as holotype; 2♀ [on slides], China, Heilongjiang Province, Hegang City, Beishan Park, 11.VII.2020, Ming-Rui Li, sweeping; 1♀ [in alcohol], China, Hebei Province, Chengde City, Mountain Wu Ling, 16.VII.2017, Guang-Xin Wang and Wen-Jian Li, sweeping.

**Figures 19–21.**
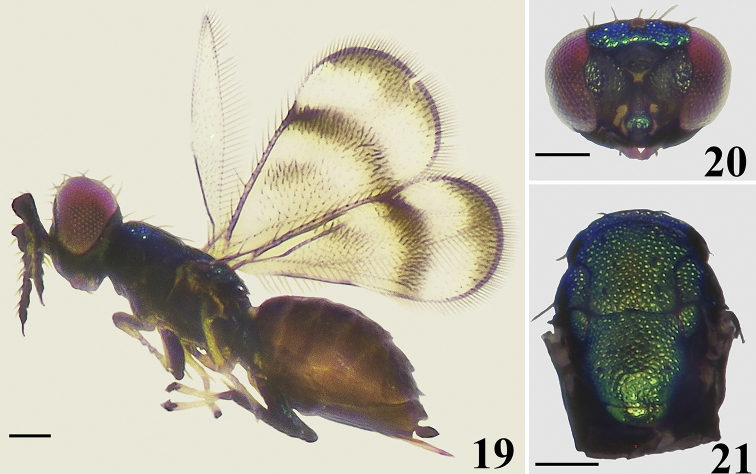
*Closterocerus separatus* Li & Li, sp. nov., holotype, female **19** habitus in lateral view, before dissection **20** head, frontal view, before slide-mounting **21** mesosoma, before slide-mounting. Scale bars: 100 μm.

##### Diagnosis.

**Female.** Head, in frontal view, oval, 1.4 times as wide as high; scape strongly compressed, and extremely expanded distad, 2.4 times as long as wide; fore wing with band I replaced by an obscure infuscate cloud; band II obvious, reaching hind margin of fore wing; band III extended along apical margin, obvious, slightly protruded inwards medially; the cloud and two bands are separated from each other; between cubital setal line and the hind margin of fore wing with a longitudinal infuscation; ventral margin of metafemur with six long setae.

##### Description.

**Female.** Length 1.06–1.15 mm. Antennae dark brown. Eyes and ocelli dull red. Frons golden-yellow to golden-green. Vertex golden-green in dorsal view, golden-blue in lateral view and frontal view. Mandibles pale brown. Pronotal collar, mesoscutum, mesoscutellum, axillae and metascutellum golden-green in dorsal view, golden-blue in lateral view. Propodeum, mesopleuron and metapleuron brown, dark brown to black. Legs with all coxae, femora and tarsal claws dark brown; pro- and mesotrochanters pale brown, metatrochanters dark brown; protibiae mainly pale yellow, pale brown basally; mesotibiae mainly pale yellow; metatibiae dark brown; all tarsi pale yellow, but first segment of metatarsi dark brown. Fore wing (Fig. 25) with band I replaced by an obscure infuscate cloud; band II obvious, reaching hind margin; band III extending along apical margin, obvious, slightly protruded inwards medially; the cloud and two bands are separated from each other; between cubital setal line and the hind margin of fore wing with a longitudinal infuscation. Metasoma dark brown with weak metallic green to blue reflections.

**Figures 22–27.**
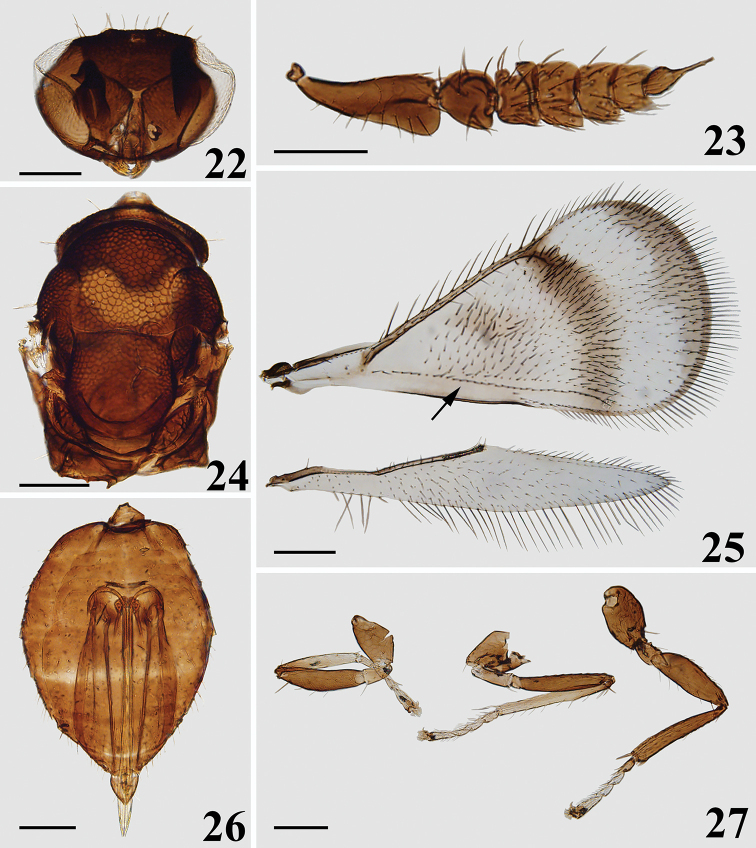
*Closterocerus separatus* Li & Li, sp. nov., holotype, female, on slide: **22** head, frontal view **23** antenna **24** mesosoma **25** fore and hind wings, arrow shows longitudinal infuscation between cubital setal line and the hind margin **26** metasoma **27** legs, from left to right: fore, mid and hind leg. Scale bars: 100 μm.

***Head*** (Figs 20, 22), oval in frontal view, 1.4 times as wide as high. Meshes of reticulate sculpture on vertex and frons below frontal sulcus smaller than that on frons above frontal sulcus. POL : OOL = 5 : 4. Frontal sulcus V-shaped, reaching eye margins; inner eye margins concave medially. Antennal scrobes join on the frontal sulcus. Malar sulcus absent, but with a curved transverse carina near clypeus, extending to lower margin of eyes. Clypeus not delimited. HE : MS : WM about 4.0 : 1.0 : 2.0. Antenna (Fig. 23) inserted above level of lower margin of eyes. Scape strongly compressed, and extremely expanded distad, 2.4 times as long as wide. Pedicel moderately compressed compared to the extremely compressed scape, nearly as long as wide. Flagellum extremely compressed; F1–4 wider than long, F2 slightly larger than F1 and F3; F3 wider than F4; F5 small, almost oval, with terminal spine long and nearly as long as the segment.

***Mesosoma*** (Figs 21, 24). Pronotum, mesoscutum, axillae and mesoscutellum (except posterior margin) with reticulate sculpture, meshes nearly of same size. Metascutellum and propodeum smooth. Along pronotal collar with four strong setae. Median area of midlobe of mesoscutum with three pairs of setae. Notauli curved in anterior part, and indicated posteriorly by depression. Mesoscutellum as long as wide. Axillae slightly advanced forwards in front of level of anterior margin of mesoscutellum. Mesoscutum and mesoscutellum rather flat. Metascutellum large, about half median length of propodeum. Propodeum without any carina in middle part. Fore wing (Fig. 25) twice as long as wide, with a stigmal hairline, radial cell bare. Speculum nearly elongate-triangular, closed below. Ratio length of: SMV : MV : PMV : STV about 5 : 11 : 1 : 2. Cubital setal line straight and completely extending to base of MV. Hind wing (Fig. 25) narrow, about 6.2 times as long as wide. Legs (Fig. 27) normal, with all coxae reticulate on outer surfaces; ventral margin of metafemur with six long setae.

***Metasoma*** (Fig. 26). Ovate; petiole short, pyriform; ovipositor exserted beyond apex of metasoma.

**Male.** Unknown.

##### Host.

Unknown.

##### Etymology.

The name refers to the separated distal two cross bands of fore wing (*separatus* is Latin for separate).

##### Distribution.

China (Heilongjiang, Hebei provinces).

##### Remarks.

*Closterocerus separatus* sp. nov. is similar to *C. africanus* Waterston, 1925, *C. cruy* (Girault, 1918) and *C. mirabilis* Edwards & La Salle, 2004, according to the original descriptions. They share the following characters with the new species: fore wing with an infuscate cloud and two infuscate bands; between cubital setal line and the hind margin of fore wing with a longitudinal infuscation; but the new species differs from *C. africanus* by having the head 1.4 times as wide as high in frontal view (1.6 times in *C. africanus*); ventral margin of metafemur with six long setae (ten setae in *C. africanus*); the area between infuscate cloud and band II of fore wing hyaline (slightly infuscate in *C. africanus*). The new species differs from *C. cruy* and *C. mirabilis* in having the infuscate cloud and two bands on the fore wing separated from each other (the cloud is distinctly connected with band II, and band III is nearly connected with band II in *C. mirabilis*; band II is distinctly connected to the cloud and bands III in *C. cruy*); apex of scape wider, about 0.4 times as wide as the length of scape (at most 0.3 times in *C. cruy* and *C. mirabilis*).

#### Closterocerus unifasciatus

Taxon classificationAnimaliaHymenopteraEulophidae

Li & Li sp. nov.

DC069D80-B37A-5DAD-B1CD-1622D9ECDD8D

http://zoobank.org/72189C1E-6C3D-4044-9242-F12985AE1701

Figs 28 , 29–35

##### Type material.

***Holotype***: ♀ [NEFU; on slide], China, Liaoning Province, Anshan City, Mountain Qian Shan, 25.VI.2015, Hui Geng, Si-Zhu Liu, Zhi-Guang Wu and Yan Gao, sweeping. ***Paratypes***: 1♀ [on slide], same data as holotype; 2♀ [1 on slide and 1 in alcohol], China, Heilongjiang Province, Yichun City, Dailing District, Liangshui Forestry Station, 29.VII.2015, Xin-Yu Zhang, Si-Zhu Liu and Xing-Yue Jin, sweeping; 1♀ [on card], China, Liaoning Province, Anshan City, Mountain Qian Shan, 23.VI.2015, Hui Geng, Si-Zhu Liu, Zhi-Guang Wu and Yan Gao, sweeping.

##### Diagnosis.

Face near clypeus with a curved, nearly V-shaped transverse carina; clypeus delimited laterally; F4 widest; fore wing with band I absent; band II becoming paler and wider posteriorly; band III extending along apical margin, much obscure (nearly imperceptible on slide); propodeum smooth and shiny, without any carina, spiracular sulcus present.

##### Description.

**Figure 28.**
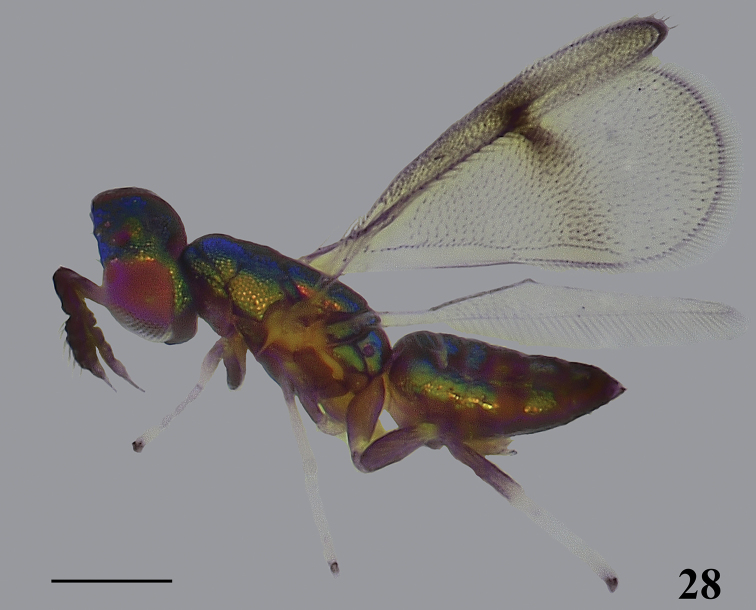
*Closterocerus unifasciatus* Li & Li, sp. nov., holotype, female, habitus in lateral view, before dissection. Scale bar: 200 μm.

**Female.** Length 0.9–1.0 mm. Scape with basal 3/5 pale brown and remainder part brown; pedicel dark brown. Funicle dark brown, clava dark brown to pale brown, becoming gradually paler from F3 to F5. Eyes and ocelli dull red. Vertex and frons above frontal sulcus golden-green, golden-blue, or golden-red. Frons below frontal sulcus golden-yellow to golden-green. Gena brown without metallic reflection. Mandibles pale brown. Pronotal collar, mesoscutum, mesoscutellum, axillae and propodeum golden-green, occasionally golden-red, in dorsal view, golden-blue in lateral view. Legs with pro- and mesocoxae pale brown, metacoxae brown; all femora and metatibiae brown, except about apical 2/7 of metatibiae pale yellow; pro- and mesotibiae mainly pale yellow, except basal part pale brown; all tarsi pale yellow and claws dark. Fore wing (Figs 28, 33) with band I absent; band II becoming paler and wider posteriorly; band III extending along apical margin of fore wing, much obscure (nearly imperceptible on slide). Metasoma concolorous with mesosoma, except median part of tergum 2 and 5, whole tergum 4, brown, with weak metallic reflections.

**Figures 29–35.**
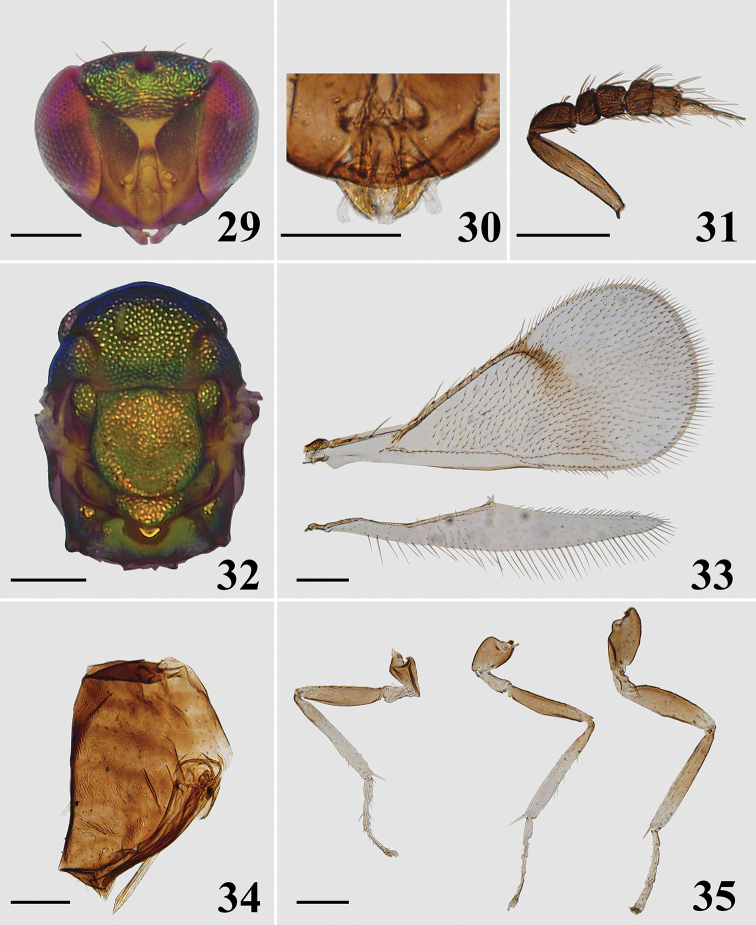
*Closterocerus unifasciatus* Li & Li, sp. nov., holotype, female, on slide (except **29** and **32**, which are photographed before slide-mounting): **29** head, frontal view **30** mandibles **31** antenna **32** mesosoma **33** fore- and hind wings **34** metasoma, lateral view **35** legs, from left to right: fore, mid and hind leg. Scale bars: 100 μm.

***Head*** (Figs 29, 30), in frontal view 1.2 times as wide as high. Vertex and frons with irregular sculpture. POL : OOL = 15 : 8. Frontal sulcus V-shaped, reaching eye margins; inner eye margins hardly concave. Antennal scrobes join on frontal sulcus. Subtorular sulci present and long. Malar sulcus absent, but with a curved, nearly V-shaped transverse carina near clypeus, extending to near lower margin of eyes. Clypeus delimited laterally. HE : MS : WM about 4.3 : 1.0 : 2.0. Antennae (Fig. 31) inserted above level of lower margin of eyes. Scape compressed, 4.8 times as long as wide. Pedicel moderately compressed compared to the compressed scape, approx. twice as long as wide. Flagellum compressed, with two funicular segments and three claval segments. F1–F3 wider than long; F2 slightly wider and longer than F1; F3, widest of all segments of antenna; F4 quadrate; F5 tapering distad, with terminal spine long and as long as the segment.

***Mesosoma*** (Fig. 32). Pronotum, mesoscutum, mesoscutellum, axillae and metascutellum with reticulate sculpture, meshes nearly of same size. Median area of midlobe of mesoscutum with two pairs of setae. Notauli curved in anterior part, and indicated posteriorly by depressions. Mesoscutellum approx. as long as wide. Axillae slightly advanced forwards in front of level of anterior margin of mesoscutellum. Mesoscutum and mesoscutellum flat. Metascutellum about 3/4 as long as median length of propodeum. Propodeum smooth, without any carina and plica, spiracular sulcus present. Fore wing (Fig. 33) slightly more than twice as long as wide, with a stigmal hairline, radial cell bare. Speculum closed below. Ratio length of: SMV : MV : PMV : STV about 5 : 10 : 1 : 2. Cubital setal line straight and completely extending to base of MV. Hind wing (Fig. 33) much narrow, about seven times as long as wide. Legs (Fig. 35) normal.

***Metasoma*** (Fig. 34). Ovate; petiole short, pyriform; ovipositor exserted beyond apex of metasoma.

**Male.** Unknown.

##### Host.

Unknown.

##### Etymology.

The name refers to the single cross band in the fore wing (*uni* is Latin for one, single and *fasciatus* is Latin for banded).

##### Distribution.

China (Heilongjiang, Liaoning provinces).

##### Remarks.

*Closterocerus unifasciatus* shares with *C. brachyphagus* Hansson, 1994 the distinct transverse band below the STV in the fore wing. The new species differs in having the mesoscutellum reticulate (smooth, without any trace of reticulation in *C. brachyphagus*); propodeum smooth, without any carina (with a weak median carina in *C. brachyphagus*) and fore wing with speculum closed below (open in *C. brachyphagus*).

## Supplementary Material

XML Treatment for Closterocerus rectisulcus

XML Treatment for Closterocerus shaanxiensis

XML Treatment for Closterocerus separatus

XML Treatment for Closterocerus unifasciatus
